# Neutrophils: Need for Standardized Nomenclature

**DOI:** 10.3389/fimmu.2021.602963

**Published:** 2021-04-15

**Authors:** Ellen McKenna, Aisling Ui Mhaonaigh, Richard Wubben, Amrita Dwivedi, Tim Hurley, Lynne A. Kelly, Nigel J. Stevenson, Mark A. Little, Eleanor J. Molloy

**Affiliations:** ^1^ Discipline of Paediatrics, Trinity College, The University of Dublin, Dublin, Ireland; ^2^ Paediatric Research Laboratory, Trinity Translational Medicine Institute (TTMI), St James’ Hospital, Dublin, Ireland; ^3^ Trinity Health Kidney Centre, TTMI, Trinity College, Dublin, Ireland; ^4^ Viral Immunology Group, School of Biochemistry and Immunology, Trinity Biomedical Sciences Institute, Dublin, Ireland; ^5^ Neonatology, Coombe Women and Infant’s University Hospital, Dublin, Ireland; ^6^ National Children’s Research Centre, Dublin, Ireland; ^7^ Viral Immunology Group, Royal College of Surgeons in Ireland‐Medical University of Bahrain, Zallaq, Bahrain; ^8^ Irish Centre for Vascular Biology, Trinity College Dublin, Dublin, Ireland; ^9^ Neonatology, Children’s Hospital Ireland (CHI) at Crumlin, Dublin, Ireland; ^10^ Paediatrics, CHI at Tallaght, Tallaght University Hospital, Dublin, Ireland

**Keywords:** neutrophils, granulopoiesis, neutrophil granules, low density neutrophils, nomenclature

## Abstract

Neutrophils are the most abundant innate immune cell with critical anti-microbial functions. Since the discovery of granulocytes at the end of the nineteenth century, the cells have been given many names including phagocytes, polymorphonuclear neutrophils (PMN), granulocytic myeloid derived suppressor cells (G-MDSC), low density neutrophils (LDN) and tumor associated neutrophils (TANS). This lack of standardized nomenclature for neutrophils suggest that biologically distinct populations of neutrophils exist, particularly in disease, when in fact these may simply be a manifestation of the plasticity of the neutrophil as opposed to unique populations. In this review, we profile the surface markers and granule expression of each stage of granulopoiesis to offer insight into how each stage of maturity may be identified. We also highlight the remarkable surface marker expression profiles between the supposed neutrophil populations.

## Introduction

Neutrophils are critical actors in the innate immune system and the body’s first line of defense against pathogens (1, 2). Approximately 100,000,000,000 neutrophils are generated in the bone marrow every day, making them the most common leukocyte in human blood (3). Neutrophils extravasate from the vasculature and are recruited to the site of infection to kill invading pathogens (4). Deficiencies in neutrophils have significant adverse effects on the overall response to infection. For instance, neutropenia, a reduction in the number of circulating neutrophils, is a condition associated with high morbidity and mortality (5). A hallmark of chronic granulomatous disease is impaired nicotinamide adenine dinucleotide phosphate (NADPH) activity, which results in reduced neutrophil bactericidal capacity (6).

The anti-microbial roles of neutrophils also include degranulation, whereby neutrophils release granule-derived mediators, engulfing pathogens by phagocytosis, and the release of neutrophil extracellular traps (NETs) which ensnare invading bacteria, fungi and viruses (7, 8). Neutrophils are highly pro-inflammatory; therefore, excessive neutrophil accumulation and prolonged activation can result in tissue damage and chronic inflammation (4). A vital activity of neutrophils is the release of reactive oxygen species (ROS). During this oxidative burst, the neutrophil consumes oxygen, which is converted to superoxide radicals *via* the NADPH oxidase 2 (NOX2) complex (4, 9, 10). Through NOX2, neutrophils primarily produce hydrogen peroxide and superoxide (11). NOX2 is highly active during neutrophil-mediated phagocytosis and localized in the phagosomal membrane to guide superoxide into the phagosome.

Neutrophils follow the leukocyte adhesion cascade to move from the bone marrow to sites of infection or inflammation (12). Novel aspects occur in the regulation of the leukocyte adhesion cascade during which time they display different phenotypes which results in various neutrophils with distinct properties (13) and specialized functions. The neutrophil response varies dramatically during this time in a process known as priming, allowing the cell to specifically target the particular site and modulate its anti-microbial action (14). This migration of neutrophils follows a circadian pattern. Circadian rhythms are important regulators of specific immune functions (15) and amongst the three main functions of neutrophils, degranulation, netosis and phagocytosis, neutrophils can produce cytokines in a circadian manner (16) fine-tuning the immune response and playing an essential role in modulating the activity of the innate immune response.

Neutrophils recognize opsonized microorganisms and engulf them *via* phagocytosis, once internalized the microorganisms are stored in intracellular vacuoles called phagosomes where they are destroyed by an oxidative burst released by active NOX2 (11). However, NETs can be produced independently of NOX2 *via* vital NETosis using mitochondrial ROS (17). NOX2 is necessary for NET production although it is unknown which oxide is involved, it is speculated to be single oxygen, superoxide, hydrogen peroxide and hypochlorous acid. NOX2 is an electron-transfer complex assembled in the plasma membrane. Gp91^phox^ is the catalytic subunit composed of the FAD-containing cytoplasmic domain, two b-type cytochromes and the p22 subunit. Neutrophil activation triggers phosphorylation events which activates enzyme activity of NOX2 by recruiting several cytoplasmic regulatory subunits to the cytoplasmic domain. Phagocytosis stimulates NOX2 assembly and electrons move through the NOX2 complex from NADPH so that oxygen loses an electron (11). The flow of electrons in NOX2 complex is in one direction, from NADPH in the cytosol to oxygen in the membrane. The pH of NOX2 phagosomes is constantly alkaline (pH 9) when the complex is active, in contrast to macrophage phagosomes.

Neutrophil dysfunction has been associated with adverse prognosis in a variety of diseases including sepsis, rheumatoid arthritis (RA), systemic lupus erythematosus (SLE), human immunodeficiency virus (HIV), mycobacterium tuberculosis infection and antineutrophil cytoplasmic antibody (ANCA)-associated vasculitis (AAV) (18–24). The production of NETs can contribute to autoimmune disease progression due to exposure to autoantigens within NETS, this occurs in several conditions such as RA, SLE and autoimmune small vessel vasculitis (25–27).

Neutrophil dysfunction is also evident in cancer whereby a high volume of intra-tumoral neutrophils is correlated with poor outcome (28). Emergency granulopoiesis typically associated with cancer progression contributes to poor patient survival, likely due to the generation of neutrophils with altered immune functions, namely immunosuppressive TANs and G-MDSC. Neutrophils contribute to the disease progression of cancer by favoring metastasis, angiogenesis and inhibiting anti-tumor immune cells, for example, inhibition of T cells *via* programmed death ligand 1 (PD-L1) (20).

The stages of neutrophil granulopoiesis are promyelocytes, myelocytes, metamyelocytes, band cells and segmented neutrophils (29). Several studies have identified a higher proportion of immature neutrophils is indicative of infection, particularly neonatal sepsis (30–32). This expansion of immature neutrophils in the bloodstream is known as *left shift* and can be measured using immature-to-total (I/T) neutrophil ratio (33) in the blood of adult patients with sepsis, the presence of immature band cells may be useful as a diagnostic marker of sepsis while immature myelocytes and metamyelocytes may be predictors of mortality (34). Immature neutrophils have been implicated in lung, breast and ovarian cancer and associated with poor prognosis (35). Therefore, the ability to differentiate neutrophil lineages is of paramount clinical importance in the setting of disease. However, it remains challenging to differentiate between stages of neutrophil granulopoiesis because there are no defined surface markers to identify immature and mature neutrophils.

Neutrophils normally have a short half-life of approximately 6-8 hours, hence studying them is a challenge as they need to be processed rapidly upon sampling (3). Measurement of absolute neutrophil counts (ANC) to detect neutrophilia and neutropenia is one of the most commonly used tests clinically (36). In this review, we propose a guide to aid in identifying the different lineages of neutrophils based on surface marker expression and correlate granule production to neutrophil function. We also explore the remarkable similarities between proposed neutrophil ‘subpopulations’.

## Neutrophil “Subset” Nomenclature

In a pre-antibiotic era, Elie Metchnikoff and Paul Ehrlich were awarded the Nobel prize in 1908 for their discovery of phagocytic cells, macrophages and neutrophils (as they were subsequently named) (37). Since this discovery of granulocytes at the end of the nineteenth century, the cells Ehrlich termed “cells with polymorphous nuclei” have been referred to by many names, including phagocytes, polymorphonuclear neutrophils (PMN), myeloid derived suppressor cells (MDSC), low density neutrophils (LDN) and tumor associated neutrophils (TANS) (1). As Shakespeare remarked “A rose by any other name would smell as sweet”, different nomenclature has been used in the literature for neutrophils in the absence of clear biological differences. This had led to the misleading concept that biologically distinct populations of neutrophils exist, particularly in disease, when in fact these are all one adaptable cell type. Neutrophils are an incredibly plastic cell type that allows them to respond and adapt to a variety of stimuli, which in fact may explain the apparent biological differences between these neutrophil “subpopulations” (38).

### Low Density Neutrophils (LDN) or Granulocytes (LDG)

Discontinuous density gradients are used to isolate neutrophils from whole blood. Normal-density neutrophils (NDN) usually reside in the high-density fraction but a subpopulation of neutrophils are found in the low-density fraction, which are known as low-density neutrophils (LDNs) or, less commonly, LD-PMN (39, 40). LDNs have been reported in a wide array of diseases including rheumatoid arthritis, systemic lupus erythematous (SLE), cancer, sepsis and asthma (41–43). LDNs display either an immature morphology with banded nuclei or myelocyte features, and are likely released in response to emergency granulopoiesis, or have a mature morphology with segmented nuclei (3, 20). Uí Mhaonaigh et al. found that CD16^int/-^CD10^-^ LDNs resemble immature neutrophils while CD16^+^/CD10^+^ LDNs share morphological features with NDNs (44). Similar to descriptions of TANs, LDNs exhibit pro-tumorigenic and immunosuppressive functions (20).

There are several theories about the origin of LDNs. Firstly, LDNs are a mixture of mature and immature neutrophils, which may be immunomodulatory (44). Secondly, LDNs could be neutrophils after undergoing degranulation and hence display a lower density which could explain the presence of neutrophils in the low density fraction (3). Interestingly, after TGF-β stimulation in a mouse model, LDNs can be derived from mature neutrophils and in human studies, LDNs can be generated by incubation with *Mycobacterium tuberculosis* (45–47). As shown in **Table 1**, G-MDSC, TANs and LDN show almost the exact same surface marker profiles. TANs and LDN also display similar pro-tumor properties, making it biologically difficult to distinguish between these subpopulations. It is possible that the plasticity of the neutrophil and the influence of the tumor microenvironment may explain how all these neutrophil “subpopulations” are in fact a normal neutrophil under the influence of a distinct local environment. There is not enough scientific evidence to confirm that G-MDSC, TAN and LDN are unique cell populations.

**Table 1:** Surface marker and functional profile of G-MDSC, TAN and LDN neutrophils.

Neutrophil Subtype	Metamyelocyte	LDN (20, 39, 41–44, 48)	TAN (canonical) (49–54)	G-MDSC (3, 55–61)
Function	Immature neutrophil subset	Pro-tumor in cancer. Present in RA, SLE, sepsis and asthma.	Pro-tumor, contributes to angiogenesis and tumor progression in cancer.	Suppress immune response and aid tumor progression in mouse model of
CD66b	+	+	+	+
CD15	+	+	+	+
CD33	+	+	+	+
CD10	–	+/-	–	–
CD11b	+	+	+	+
CD16	+	+/-^int^	+	+
HLA-DR	–	+	–	–
CD62L	–	?	+	+
CXCR2	+	?	+	+
CXCR4	+	?	+	+

Cassetta et al. suggest that information in the literature regarding neutrophil subsets, such as G-MDSC, LDN, Tan, is varied and contradictory due to the use of different models and isolation techniques. Surface markers on neutrophils isolated from murine models and non-human primates correlate poorly with corresponding human markers. Not only are cell surface markers different between mice and humans but some neutrophil subtypes, such as MDSC, are isolated from blood in humans but are studied at tissue level in mice (58). Therefore, standardized protocols are essential to gain further insight into the biological significance of neutrophil subtypes.

### Myeloid Derived Suppressor Cells (MDSC)

MDSC were identified as myeloid cells that suppress immune responses and aid tumor progression in mouse models of cancer but not in humans (55, 62). The cells were named MDSC by Gabrilovich et al. (63). There are believed to be two subpopulations of MDSC: monocytic (Mo-MDSC) and granulocytic (G-MDSC or polymorphonuclear (PMN)-MDSC) (64, 65). Pillay et al. suggest that G-MDSC are a *bona fide* phenotype of neutrophils, which (unlike conventional neutrophils) are found in the low density fraction of peripheral blood (3, 64). Mo-MDSC are also found in the low density layer and are morphologically similar to monocytes (20). Interestingly, G-MDSC show similar morphology to mature neutrophils (66). MDSC display similar surface marker expression patterns to neutrophils but it is their suppressive functions which define this population: cluster of differentiation molecule (CD)66b^+^, CD16^+^, CD15^+^ and CD14^-^ (56). As reviewed by Rosales in 2018, both Mo-MDSC and G-MDSC are low density, CD11b^+^, CD33^+^ and CD66b^+^, G-MDSC are human leukocyte antigen-DR isotype (HLA-DR)^-^ and Mo-MDSC are HLA-DR^-/low^ (3). Mo-MDSC differ from G-MDSC in being CD14^+^ and CD15^-,^ whereas G-MDSC are CD14^-^ and CD15^+^ (3, 57). G-MDSC are often further subdivided based on whether they are CD16^-^ or CD16^+/INT^ (56). G-MDSC are most likely a mixed low-density population of immature and mature neutrophils. A general consensus has been established for identification of G-MDSC whereby there must be, at a minimum, the following surface marker profile: CD15^+^, CD11b^+^, CD14^-^, HLA-DR^-^, and CD33^mild^ (58).

### Tumor-Associated Neutrophils (TAN)/N2

Tumor-associated neutrophils (TAN) may be polarized towards two potential phenotypes similar to macrophages: N1, which are anti-tumor, and N2, which are pro-tumor, however, these are limited to mouse models and not yet identified in humans. Each subpopulation has distinct functions, cytokines and gene expression profiles (50, 67). N2 have circular nuclei while N1 show hypersegmented nuclei (49). N2 are promoted by transforming growth factor-β (TGF-β) and N1 are recruited by interferon-β (IFN-β), N1 neutrophils are likely stimulated by the tumor microenvironment (49, 50, 68). Tumor cells use chemokines to attract TANs to the tumor site, such as the potent neutrophil chemoattractant CXCL8, which entrains the CXCR1 and CXCR2 expression on neutrophils. CXCR1 has been shown to contribute to angiogenesis and tumor progression (50, 69). TANs are believed to be distinct from normal density neutrophils (NDN) and G-MDSC as TANS exhibit high chemokine production, few granules and low ROS production (70). TANs may be evidence of the plasticity of neutrophils in response to factors in the tumor microenvironments of specific cancers, rather than a novel neutrophil subpopulation (20).

## Neutrophil Granulopoiesis Stages Defined by Density, Morphology and Maturity

### Maturity

Neutrophils are a heterogenous population comprised of phenotypically distinct subtypes during granulopoiesis (71). To date, two contrasting models have been described for the generation of blood cells from hematopoietic stem cells (HSC). These are the ‘classical model’ that has been used for generations and describes a cells ability to determine its cellular fate prior to single lineage commitment and is subsequently defined by its inability to differentiate into other progenitor cells. This model describes HSCs giving rise to either Common myeloid progenitors (CMPs) or common lymphoid progenitors (CLPs). The CMP then further differentiates into either a granulocyte monocyte progenitor (GMP) or a megakaryocyte erythroid progenitor (MEP). However, with the recent advancements in single cell sequencing, an ‘alternative model’ has been proposed which highlights that both CMPs and CLPs have mixed lineage potential defined by their transcription heterogeneity and their cellular fate is determined by external differentiation factors (72).

Transcription factors have long been known to regulate the commitment and subsequent activation of various myeloid derived cells from HSCs (73). This complex process involves the upregulation of and silencing of various developmental genes under the control of certain transcription factors (TF). The process that governs the development of CMPs to GMPs is dependent on the following transcription factors including CCAAT/enhancer-binding proteins (C/EBPs), GATA-1, and PU.1 (74). C/EBP-α, -β, and –ϵ have long been known to regulate neutrophil development in which mutations in either result in the development of myeloid leukemia (75, 76). Additional transcription factors including PU.1, and Irf8 induce CMPs to differentiate into monocytes whereas neutrophil differentiation involves a complex interplay of transcription factors Gfi-1, PU.1 and C/EBPs (77).

**Figure 1:**
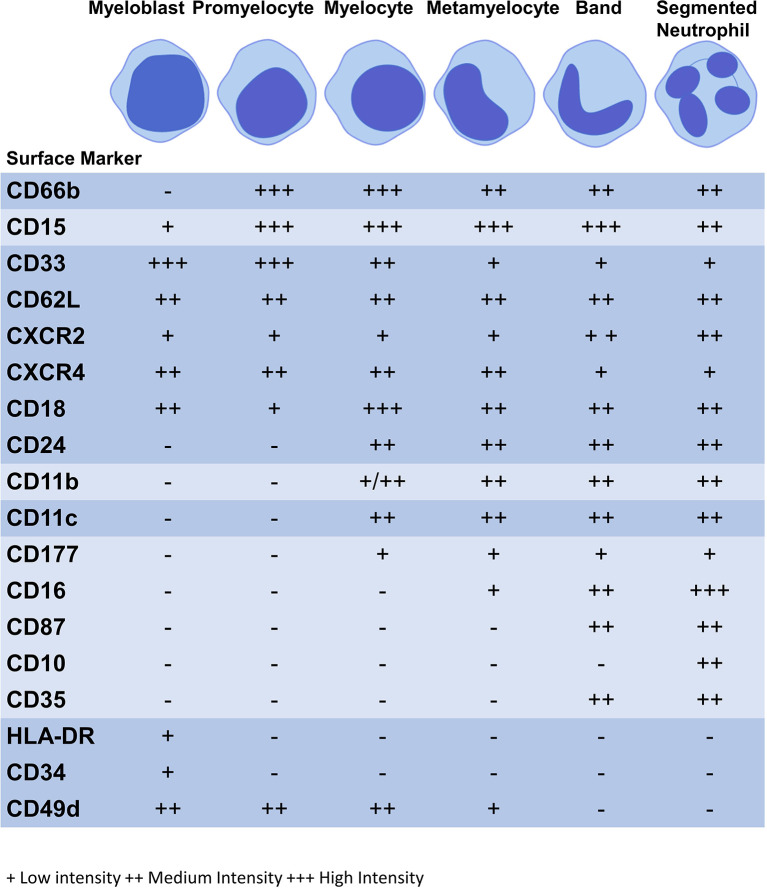
Expression of surface markers during granulopoiesis. Figure illustrates the surface marker expression at each stage of granulopoiesis; myeloblast, promyelocyte, myelocyte, metamyelocyte, band cell and segmented neutrophil. The intensity of the surface marker is shown whereby; low intensity (+), medium intensity (++) and high intensity (+++).

In the bone marrow, hematopoietic stem cells (HSCs) differentiate into myeloblasts, which in turn become promyelocytes, myelocytes, metamyelocytes, band cells and, lastly, segmented neutrophils (29) (**Figure 1**). Inside the bone marrow there are three compartments where neutrophils reside: stem cell pool, mitotic pool and post-mitotic pool (78). Undifferentiated progenitor cells such as HSCs, are found in the stem cell pool, the mitotic pool holds myeloblasts, promyelocytes and myelocytes and, finally, metamyelocytes, band cells and segmented neutrophils are localized within the post-mitotic pool (33). At the metamyelocyte stage, the neutrophil can no longer proliferate, signaling the start of terminal differentiation (79). In the absence of infection, there are more neutrophils stored in the bone marrow than in the circulation. In response to signals at the site of infection, only mature segmented neutrophils migrate out of the bone marrow in large numbers to the site of infection (80).

Terminal differentiation of GMPs into neutrophils involves the acquisition of neutrophil specific granule components at various stages of neutrophil maturation. This includes the storing of primary granules such as MPO and NE (at early stages), and, subsequently, Cathelicidin and lactoferrin (81). An important TF in this process is lymphoid enhancer factor-1 (Lef-1), deficiency of which results in impaired neutrophil maturation through the expression *C/EBPα* (82). Moreover TF Erg, Myb, Fos-like antigen (Fosl) 1, Fosl2, JunB proto-oncogene, B-cell lymphoma (Bcl) 6, Kruppel like factor (Klf) 6, and interferon regulatory factor 1 (Irf) 1 are known to regulate late stage neutrophil differentiation, revealing a complex network of TF involved in the process of granulopoiesis (83).

## Neutrophil Density and Its Association With Neutrophil Development

The increased granularity and cell size that occurs with neutrophil maturity is directly proportional to a change in density. Therefore, neutrophil lineages can be separated using density gradient centrifugation (35). Mature neutrophils localize in normal to high density fractions, while immature neutrophils reside in low density fractions, although this is probably preparation dependent and the low density fraction is likely a mixed population of neutrophil maturity (35, 46).

### Morphology

Neutrophil subtypes differ in their nuclear morphology. The most immature neutrophils, the myeloblasts, have large, sphere-shaped nuclei containing few nucleoli. Promyelocytes and myelocytes lack nucleoli and exhibit elevated chromatin condensation compared to myeloblasts. An indented nucleus is characteristic of a metamyelocyte. The band cell nucleus is shaped like a horseshoe and it constricts to form nuclear lobes, while segmented neutrophils are distinguishable by their segmented nucleus, with three to five lobes (84). The thin filaments which connect the lobes contribute to the migratory capability of mature neutrophils (85).

## Neutrophil Surface Marker Expression During Granulopoiesis

Neutrophil surface markers change to facilitate altered functions as the neutrophil matures (**Table 2**). During this process, immature surface markers are no longer be expressed (e.g. CD49d) and markers of maturity appear (e.g. CD10) (86). Although there is no consensus on human neutrophil phenotypic markers on flow cytometry the following are commonly used: CD11b^+^CD66b^+^CD15^+^CD14^-^ (20). CD16 is a marker of phagocytic capacity and may be used to exclude CD16^-^ eosinophils. CD14^+^ cells are considered to be mostly macrophages and monocytes, although some studies indicate that neutrophils express CD14 at low levels (115, 116). CD16 is exclusively expressed at the metamyelocyte stage and is highly expressed by banded and segmented neutrophils, while activation marker CD11b is only found at the myelocyte stage onwards (94, 97–100). CD11b and CD18 form the Mac-1 complex which plays a role in phagocytosis and migration with CD18 expression commencing at the myeloblast stage of granulopoiesis (86). Differentiation marker CD15 and activation marker is found on all neutrophil subpopulations and CD66b from the promyelocyte stage; these are also core markers of neutrophil lineage (86–88). Surface markers may be expressed at low, medium or high intensities, see **Figure 1** (86, 88).

**Table 2:** Neutrophil surface marker expression.

Surface marker	Protein name	Surface marker type	Lineage stage	Reference
CD66b	Carcinoembryonic antigen-related cell adhesion molecule 8 (CEACAM8)	Granulocyte activation marker and neutrophil lineage marker	Promyelocyte-segmented neutrophil	(86–89)
CD15	Lewis^x^, X-hapten	Differentiation marker and neutrophil lineage marker	Promyelocyte-segmented neutrophil	(86, 90, 91)
CD33	Gp 67	Differentiation marker	Myeloblast-segmented neutrophil	(91–94)
CD49d	VLA-6 α subunit, α5 integrin subunit	Adhesion marker	Myeloblast-metamyelocyte	(95–97)
CD10	Common acute lymphoblastic leukemia antigen (CALLA)	Differentiation marker of maturity	Segmented neutrophil	(87, 98)
CD11b	Complement receptor 3, integrin αM subunit, Mac-1	Phagocytosis, part of the Mac-1 complex with CD18, activation marker	Myelocyte- segmented neutrophil	(94, 97–102)
CD11c	Complement receptor 4, integrin αX subunit	Cell migration	Myelocyte- segmented neutrophil	(94, 103)
CD18	Integrin β_2_ subunit	Phagocytosis, part of the Mac-1 complex with CD11b	Promyelocyte-segmented neutrophil	(101)
CD34	Unknown	Adhesion marker and marker of progenitor neutrophil cells and hematopoietic stem cells	Myeloblast	(94, 104)
CD16	FcgammaR3b	Marker of phagocytotic capacity	Metamyelocyte- segmented neutrophil	(35, 97–99)
HLA-DR	MHC class II	Antigen presentation to CD4^+^ T cells.	Myeloblast. Not expressed on circulating neutrophils but is found on tissue neutrophils under inflammatory conditions, such as RA synovial fluid	(94, 105–107)
CD24	Heat-stable antigen (HSA), BA-1	Differentiation marker	Myelocyte-segmented neutrophil	(97)
CD87	Urokinase plasminogen activator receptor (uPAR)	Cell migration	Band-segmented neutrophil	(98)
CD35	Complement receptor 1	Adherence of C4b and C3b-bound ligands after internalization	Band- segmented neutrophil	(97, 98, 108)
CD62L	L-selectin	L-selectin involved in adhesion	Myeloblast-segmented neutrophil	(109, 110)
CXCR2	Interleukin 8 receptor	Neutrophil mobilization and exit from bone marrow	Myeloblast- segmented neutrophil	(73, 86, 88, 111, 112)
CXCR4	CXC chemokine receptor type 4	Neutrophil retention in /return to bone marrow	Myeloblast-segmented neutrophil	(73, 88, 113)
CD177	Neutrophil specific antigen 1 (NB1)	Extravasation	Myelocyte-segmented neutrophil	(114)

## Markers of Maturity and Immaturity

HLA-DR (major histocompatibility complex II, MHC class II), which is involved in antigen presentation to CD4^+^ T cells, and CD34, an adhesion marker, are two markers exclusively expressed on the most immature neutrophil, the myeloblast (94, 106). HLA-DR is not present on circulating neutrophils but is expressed on the surface of tissue neutrophils under specific inflammatory conditions, such as RA synovial fluid (105). CD33 is a differentiation marker found on myeloid blast cells in acute myeloid leukemia (AML) (93). CD33 surface expression is gradually downregulated from the myeloblast stage to segmented neutrophil, with a low level expressed on the latter (91, 92).

CD10, a marker of differentiation and maturity is found only on mature segmented neutrophils, and absent on immature neutrophils. Immature CD10^-^ neutrophils may be important drivers of inflammatory disease and neutropenia (87, 88, 98). Reduced surface expression of CD10 and CD16 on granulocytes predicts poor outcome in sepsis patients (117). CD10^dim^ immature neutrophils have been implicated in the immunosuppression observed in sepsis (118).

Conflicting results exist for the marker of maturation CD24. Elghetany et al. found that CD24 expression begins at the myelocyte stage and is a marker of maturation (97). In contrast, Hernández-Campo et al. found that CD24 is present on CD34^-/low^ myeloblasts, is highly expressed by myelocytes and decreases from metamyelocyte to segmented neutrophil (119).

## Markers of Adhesion and Extravasation

The integrin very late antigen complex 4 (VLA-4) is involved in the adhesion of hematopoietic progenitor cells and leukemic blast cells in AML (120). VLA-4 is composed of CD49d and CD29, CD49d being a commonly used marker of neutrophil immaturity (120, 121). Promyelocytes, myelocytes and metamyelocytes are CD49d^+^ while more mature bands and segmented neutrophils are CD49d^-^ (97). Given the role of CD49d in the recruitment of progenitor cells leukemic blast cells and their migration from the bone marrow, it is likely that CD49d must be expressed on neutrophil blast cells (95, 96). Interestingly, CD49d expression reappears on the surface of aged neutrophils (122).

High CD162, or P-selectin glycoprotein ligand-1 (PSGL1), is an L-selectin molecule involved in adhesion that is found on the surface of myeloblasts to segmented neutrophils (109, 123). CD35, involved in the adherence of C4b and C3b bound ligands after internalization, and CD87, involved in cell migration, are solely expressed by bands and segmented neutrophils (97, 98). CD11c is a another marker of cell migration and is expressed at the myelocyte stage (94, 103).

Surface expression of CD177, a receptor involved in extravasation and surface expression of proteinase-3, begins at the myelocyte stage (114). Interestingly, there is an increase in CD177^+^ circulating neutrophils in diseases such as sepsis, vasculitis and SLE (124, 125).

## Chemokines and Chemokine Receptors

Immature neutrophil retention in the bone marrow is achieved by the cross-talk between CXCR4 expressed on neutrophils and CXCL12 by bone marrow stromal cells. CXCR4 is involved in neutrophil retention in the bone marrow and return of aged neutrophils to the bone marrow (73, 126). Upregulated expression of CXCR4 on segmented neutrophils ready to undergo senescence or apoptosis triggers their return to the bone marrow where they are engulfed by macrophages (113, 127). While both immature and mature neutrophils express CXCR4, immature neutrophils may express a higher level of CXCR4 than mature cells (73, 88). The chemokine receptor CXCR2 plays a role in neutrophil mobilization and exit from the bone marrow (86, 111). Immature neutrophils before the band stage exhibit reduced CXCR2 surface expression compared to bands and segmented neutrophils (73, 88). However, CXCR4 and CXCR2 may not be useful in differentiating between neutrophil subtypes due to similar expression levels in each population.

Thus, surface markers on neutrophils demonstrates that flow cytometry, or other techniques, could be employed to separate neutrophil lineages based on surface marker expression. This information highlights that banded neutrophils and segmented neutrophils are the most similar in their surface expression and may be the most difficult to differentiate due to a lack of unique markers on either cell type (110, 128). Some surface markers undergo changes following migration, e.g. CD62L and activation, e.g. CD62L/CD11b/CD18, which makes it difficult to identify highly specific markers of maturity (35).

## Neutrophil Granules

**Figure 2:**
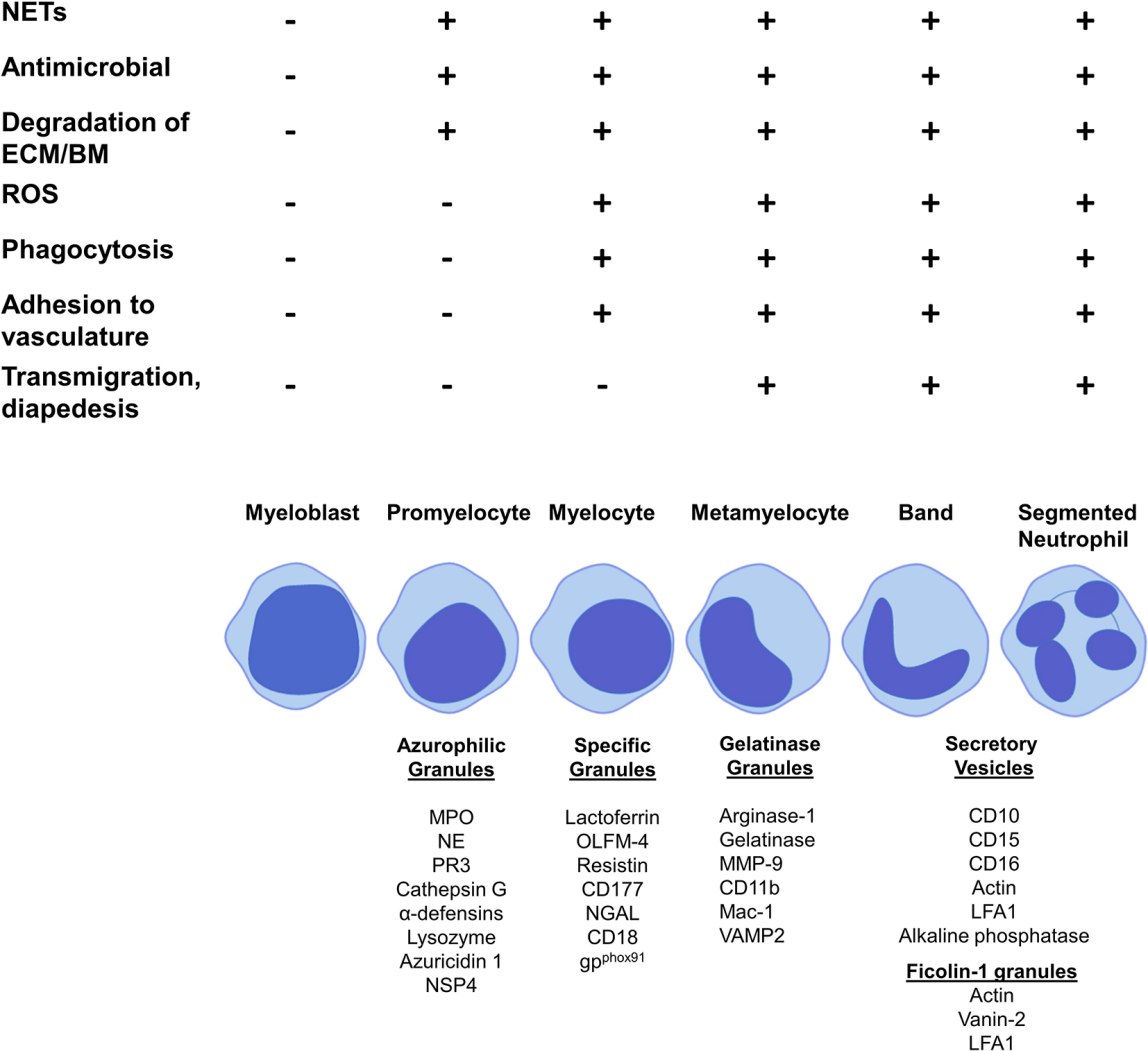
Granule production and functionality during granulopoiesis. Figure shows granule production during the stages of granulopoiesis. Granule formation begins with azurophilic granules at the promyelocyte stage, specific granules are first formed by myelocytes, gelatinase granules are formed at the metamyelocyte stage and only band cells and segmented neutrophils can form ficolin-1 granules and secretory vesicles. NET formation, antimicrobial functions and degradation of the extracellular membrane and basement membrane begin at the promyelocytes stage with azurophilic granule formation. ROS production, phagocytosis and adhesion to vasculature starts at the myelocytes stage with the formation of specific granules. Metamyelocytes are the first stage to perform transmigration and diapedesis through a vessel wall, which correlates with gelatinase granule formation.

The production of neutrophil granules begins as the immature neutrophil transitions from a myeloblast to a promyelocyte and continue to be produced up to the segmented neutrophil stage (129). There are four main groups of granules: azurophilic (primary) granules, specific (secondary) granules, gelatinase (tertiary) granules and (most recently discovered) ficolin-1 granules (**Figure 2**, **Table 3**). Neutrophils also contain secretory vesicles, which are not defined as granules (101, 144). A comprehensive review of granulopoiesis granule production and associated transcription factors is provided by Lawrence et al. (82).

**Table 3:** Functionality and localization of neutrophil granules.

Protein	Key function	Granule	Lineage stage of onset	Reference
**MPO**	MPO uses hydrogen peroxide to generate secondary oxidants necessary to destroy pathogens. MPO also plays a role in NETosis	Azurophilic granules	Promyelocyte	(11, 82, 130, 131)
**Neutrophil elastase**	Roles in NETosis, adhesion, ECM degradation. Plays roles in non-oxidative pathways of destroying pathogens, both intracellular and extracellular	Azurophilic granules	Promyelocyte	(129–133)
**Cathepsin G**	NETosis, adhesion, ECM degradation. Plays roles in non-oxidative pathways of destroying pathogens, both intracellular and extracellular	Azurophilic granules	Promyelocyte	(129, 130, 132–134)
**Proteinase 3**	NETosis. Plays roles in non-oxidative pathways of destroying pathogens, both intracellular and extracellular	Azurophilic granules	Promyelocyte	(82, 129, 130, 132, 133)
**Lysozyme**	Killing of bacteria by hydrolysis of cell wall peptidoglycan, ECM degradation	Azurophilic granules	Promyelocyte	(129, 132, 135)
**NSP4**	ECM degradation	Azurophilic granules	Promyelocyte	(82, 129, 132)
**Azurocidin 1**	Antibacterial functions	Azurophilic granules	Promyelocyte	(82, 129, 132)
**Alpha-defensins**	Anti-microbial functions and roles in NETosis	Azurophilic granules	Promyelocyte	(130, 131, 136)
**Flavocytochrome b (gp^91phox^)**	Phagocytosis	Specific granules	Promyelocyte	(137)
**Lactoferrin**	Role in NETosis and anti-bacterial properties.	Specific granules	Myelocyte	(129, 132)
**OLFM-4**	Bacterial infections against *S. aureus*	Specific granules	Myelocyte	(82, 138)
**Resistin**	Chemoattractant	Specific granules	Myelocyte	(82, 139)
**CD177**	Adhesion, extravasation, antimicrobial functions	Specific granules	Myelocyte	(82, 140–142)
**NGAL**	Antimicrobial functions	Specific granules	Myelocyte	(82, 143)
**MMP-9**	Migration through extracellular matrix	Gelatinase granules	Metamyelocyte	(81, 129, 132)
**Gelatinase**	Migration through basement membrane	Gelatinase granules	Metamyelocyte	(81, 129, 132)
**Mac-1**	Phagocytosis, adhesion, crawling, transmigration and diapedesis of vessel wall	Gelatinase granules	Metamyelocyte	(101, 129, 145)
**LFA1**	Rolling, adhesion, transmigration and diapedesis of vessel wall	Secretory vesicles and ficolin-1 granules	Band	(82, 144, 145)
**VLA-4**	Rolling, tethering, adhesion, crawling, transmigration and diapedesis of vessel wall	Granule unknown	Unknown	(145)

Prominent production of the heme enzyme myeloperoxidase (MPO) is the main characteristic feature of azurophilic granules. MPO makes up 5% of the weight of a neutrophil (82). **Azurophilic granules** contain MPO, neutrophil elastase (NE), proteinase 3 (PR3), lysozyme, azurocidin 1, cathepsin G and neutrophil serine protease 4 (NSP4) (129, 132, 133). The main neutrophil antimicrobial peptides, α-defensins, are also stored in azurophilic granules (136). The formation of azurophilic granules begins at the promyelocyte phase (132) (**Figure 2**). Cathepsin G and NE may contribute to downregulation of neutrophil adhesion by proteolysis of CD62L, preventing its binding to P-selectin on the endothelium (146). Lysozyme mediates killing of bacteria by hydrolyzing the peptidoglycan cell wall (135). NE, PR3, MPO, α-defensins, and cathepsin G are all involved in NETosis (130, 131, 147–149). PR3, cathepsin G, NE and NSP4 contribute to extracellular matrix (ECM) degradation by mediating proteolysis of components such as laminin, elastin and type IV collagen (82).

**Specific granules** contain large amounts of lactoferrin, which plays a key role in NETosis (129, 132). Other components are olfactomedin-4 (OLFM-4), which helps fight bacterial infections such as *Staphylococcus aureus*, resistin, a pro-inflammatory cytokine and T-cell chemoattractant, the receptor CD177 and antimicrobial neutrophil gelatinase-associated protein lipocalin (NGAL) (82, 138–140, 143). Specific granules are formed at the myelocyte stage (82) (**Figure 2**).

**Gelatinase granules** store arginase 1, matrix metallopeptidase (MMP)-9, gelatinase and surface receptors including CD11b/CD18 (Mac-1) (129, 132). The main function of gelatinase granules such as MMP-9 and gelatinase is to facilitate the breakdown of the ECM and basement membrane, aiding migration into tissue (81). Metamyelocytes begin to form gelatinase granules (101) (**Figure 2**).

Segmented neutrophils form **ficolin-1 granules**, the contents of which play roles in migration and adhesion, for example, actin and vanin-2 (82, 144). Segmented neutrophils also produce secretory vesicles, these contain actin, alkaline phosphatase and receptors including CD11b, CD10 and CD16 (82, 101, 144) (**Figure 2**).

## Neutrophil Granules Involved in Neutrophil Extracellular Traps

Active neutrophils undergo NETosis, a form of cell death that involves releasing decondensed chromatin in response to stimuli, such as bacteria (7). NETs contribute to a wide range of inflammatory diseases including as rheumatoid arthritis, systemic lupus erythematosus and autoimmune small vessel vasculitis (25–27). NETs exhibit anti-microbial functions by trapping extracellular microbes (128). The formation of NETs in response to pathogens involves key granular components: NE, PR3, MPO, α-defensins, cathepsin G, lactoferrin, and reactive oxygen species (ROS) (130, 131, 147–149). NETs are induced by the translocation of NE from granules to the nucleus where it cleaves histones, resulting in chromatin decondensation and breakdown of the plasma membrane (150). With the exception of lactoferrin, which is located in specific granules, all other major proteins involved in NET formation are located in azurophilic granules whose formation begins at the promyelocyte stage. Therefore, NET formation likely begins at this stage of granulopoiesis (129, 132) (**Figure 2**). To our knowledge, no study has identified any surface markers of NETosis.

## Neutrophil Granules Involved in ROS Production and Phagocytosis

Neutrophils are the greatest producers of ROS in response to infection. The NADPH oxidase complex is responsible for the production of ROS, which drives the antimicrobial function of neutrophils by inducing degranulation, NETosis and release of pro-inflammatory cytokines (151). Phagocytosis is the process of engulfing and destroying pathogens (152). The NADPH oxidase complex localizes in the phagocytic vacuole and causes a ‘respiratory burst’ of oxygen, producing superoxide (O_2_-) during phagocytosis. A flavocytochrome b, gp91^phox^, enables electron transfer and interacts with NADPH in the phagocytic vacuole (153). CD11b/CD18 (Mac-1) and flavocytochrome b are formed and stored in gelatinase and specific granules, indicating that these granules play a role in phagocytosis (101, 129, 137). This implies that phagocytosis begins at the myelocyte stage when these specific granules are formed (129, 132) (**Figure 2**).

## Neutrophil Granules Involved in Degranulation

The steps involved in neutrophil degranulation are, firstly, translocation of granules to the target membrane, which is achieved by the assembly of microtubules and actin rearrangement. G protein, Rab, and snap receptor (SNARE) membrane trafficking proteins facilitate docking of granules which can then be released from the neutrophil by exocytosis (154). SNARE protein vesicle-associated membrane protein 2 (VAMP2) aids the exocytosis of granules from the neutrophil and is formed in gelatinase granules (8, 101). Granules produced at more advanced stages of neutrophil granulopoiesis are more likely to be released by exocytosis than those formed in early stages. Degranulation appears to occur at a low level with the formation of azurophilic granules by promyelocytes and increases with each subsequent stage of granulopoiesis, with secretory vesicles produced by bands and segmented neutrophils accounting for the highest degree of degranulation (154, 155) (**Figure 2**).

## Neutrophil Granules Involved in Extravasation

Neutrophil extravasation is the process by which circulating neutrophils pass through the blood vessel endothelium to reach the site of infection. The neutrophil extravasation cascade is initiated by leukocytes at the site of infection, which release inflammatory signals (e.g. histamine) to induce changes in endothelial cells, for instance the upregulation of P-selectin. Extravasation is facilitated by receptor-ligand interactions between the neutrophil and endothelial cells. The steps involved include neutrophil tethering to endothelial cells *via* receptor-ligand interactions, adhesion to the endothelium, crawling, rolling and, finally, transmigration through gaps between endothelial cells (145).

CD177 has a high affinity for platelet endothelial cell adhesion molecule (PECAM-1), allowing the neutrophil to pass through the endothelium (141, 142). CD177 and PR3 are co-expressed on the neutrophil surface and may co-operate to promote extravasation, although a recent study has indicated that CD177 may have an inhibitory effect on PR3 (141, 156). CD177 is stored in specific granules starting at the myelocyte stage and is expressed on the surface of myelocytes through to segmented neutrophils (114, 140).

Mac-1 (CD11b/CD18) plays a vital role in neutrophil extravasation. It binds to endothelial markers ICAM1 and ICAM2 and facilitates neutrophil adhesion, crawling along the blood vessel wall, transmigration and diapedesis (145). The Mac-1 complex can only be formed when both CD11b and CD18 have been produced; CD18 is produced by promyelocytes in specific granules and CD11b is produced by myelocytes in gelatinase granules. Therefore, Mac-1 is first formed at the myelocyte stage (101) (**Figure 2**).

Leucocyte function antigen 1 (LFA1) is composed of CD11a and CD18 and is involved in neutrophil rolling and adhesion by binding to ICAM1 and transmigration and diapedesis *via* binding to junctional adhesion molecule A (JAM-A) on the endothelium (145). LFA1 is stored in secretory vesicles and ficolin-1 granules and, therefore is only produced by bands and segmented neutrophils (82, 144).

There are several other adhesion molecules involved in neutrophil extravasation with unclear granule location; these are β2-integrin, L-selectin (CD62L), CD44 and CD49. In terms of extravasation, VLA-4 (CD49D/CD29) aids neutrophil tethering, rolling, adhesion, transmigration and diapedesis *via* VCAM1 or JAM-B. Interestingly, VLA-4 is produced by myelocytes but the specific granule that produces VLA-4 is unknown (145).

## Conclusions

The neutrophil is an adaptable cell type capable of rapidly responding to changes in its environment. However, the neutrophil has been given many names in the literature, suggesting that multiple unique populations exist despite biological similarities. There is a lack of standard techniques for isolating neutrophils, which may account for varied results in the literature, and differences between these neutrophil populations. Neutrophils from murine models and humans have distinct patterns of surface markers that make it difficult to draw significant conclusions about the idiosyncratic nature of neutrophil populations such as MDSC, LDN and Tan. Therefore, standardized protocols are necessary to gain further insight into the biological significance of neutrophil populations and determine whether they are genuine, distinct populations or a result of activation during isolation or differences in the biology of mice and humans.

Different neutrophil nomenclature is often necessary, for instance LDN and NDN to differentiate between neutrophils of different densities and since LDNs are often present at higher levels in disease. Minimal nomenclature based on biological properties such as density should be maintained. We suggest using the longstanding nomenclature describing mature and immature neutrophils with immature types classified as band forms, myelocytes, promyelocytes and metamyelocytes with clarification of the surface markers for each on flow cytometry. This can then standardize the cell types across all disease states rather than the plethora of names such as MDSC, LDN and TAN often describing similar cells. The detailed function of each cell stage of maturity, granules and surface markers could be clarified. This would allow targeted interventions in distinct cell types that could be used in areas from cancer to other inflammatory disorders. In the future which would allow standardized classification and allow more extensive collaboration to characterize all functional and phenotypic variation among all the cells with this classification system.

## Author Contributions

EM, AU, and RW conducted the research and wrote the manuscript. EJM supervised the work and co-wrote and edited the manuscript. AD, TH, LK, NS, and ML assisted in writing and revision of the manuscript. All authors contributed to the article and approved the submitted version.

## Conflict of Interest

The authors declare that the research was conducted in the absence of any commercial or financial relationships that could be construed as a potential conflict of interest.
